# Analysis of the Polycomb-related lncRNAs *HOTAIR* and *ANRIL* in bladder cancer

**DOI:** 10.1186/s13148-015-0141-x

**Published:** 2015-10-08

**Authors:** Mónica Martínez-Fernández, Andrew Feber, Marta Dueñas, Cristina Segovia, Carolina Rubio, Maria Fernandez, Felipe Villacampa, José Duarte, Fernando F. López-Calderón, Ma José Gómez-Rodriguez, Daniel Castellano, Jose L. Rodriguez-Peralto, Federico de la Rosa, Stephan Beck, Jesús M. Paramio

**Affiliations:** Molecular Oncology Unit, CIEMAT (ed70A), Av Complutense 40, 28040 Madrid, Spain; Biomedical Research Institute I+12, University Hospital “12 de Octubre”, Av Córdoba s/n., 28041 Madrid, Spain; Medical Genomics, UCL Cancer Institute, University College London, Paul O’Gorman Building, 72 Huntley Street, London, WC1E 6BT UK; Servicio de Anatomía Patológica, Hospital Universitario 12 de Octubre, Instituto de Investigación 12 de Octubre i+12, UCM, Av Cordoba s/n., 28041 Madrid, Spain

**Keywords:** Epigenetics, Bladder cancer, LncRNA, HOTAIR, Recurrence, ANRIL

## Abstract

**Background:**

Long non-coding RNAs (lncRNAs) have been claimed as key molecular players in gene expression regulation, being involved in diverse epigenetic processes. They are aberrantly expressed in various tumors, but their exact role in bladder cancer is still obscure. We have recently found a major role of the Polycomb repression complex in recurrence of non-muscle-invasive bladder cancer. Here, we report the xpression of Polycomb-related lncRNAs:antisense noncoding RNA in the INK4 locus *(ANRIL)* and HOX antisense intergenic RNA (*HOTAIR*) in these tumors.

**Findings:**

We studied a dataset of non-invasive bladder cancer samples by quantitative reverse transcription PCR (RT-qPCR) and analyzed also invasive bladder cancer samples using TCGA data. Our results showed that, while *ANRIL* seemed not to have a determining role, an increased *HOTAIR* expression appeared in recurrent and high-graded tumors associated with poor prognosis. In addition, through genome-wide transcriptome analyses, we observed that *HOTAIR-EZH2*-complex-regulated genes can efficiently discriminate between non-tumoral, recurrent, and non-recurrent bladder cancer samples. We also observed a significant correlation between EZH2 and *HOTAIR* expression levels. Using overexpression, knockdown, and pharmacological approaches in bladder cancer cell lines, we also observed that EZH2 regulates *HOTAIR* expression.

**Conclusions:**

Our findings indicate that *HOTAIR* expression has prognostic value for bladder cancer progression, recurrence, and survival and suggest that *HOTAIR* plays active roles in modulating the cancer epigenome, becoming an interesting candidate as a target for cancer diagnosis and therapy. The observed *HOTAIR* regulation by EZH2 and the possibility of modulating EZH2 activity with specific inhibitors open new possible paths to be explored in bladder cancer therapy.

**Electronic supplementary material:**

The online version of this article (doi:10.1186/s13148-015-0141-x) contains supplementary material, which is available to authorized users.

## Findings

### Background

In the past decades, little progress has been achieved in the development of biomarkers for bladder cancer (BC), in spite of its high prevalence and of being a leading cause of cancer-associated deaths in developed countries. At diagnosis, 75 % of cases are categorized as superficial tumors, Ta or T1 (localized in the mucosa or submucosa, referred as non-muscle-invasive bladder cancer: NMIBC) while 25 % are already invasive cancer, T2–T4 (muscle-invasive bladder cancer: MIBC). In the case of NMIBC, recurrences are frequent, requiring continuous surveillance and becoming one of the most costly cancers to treat. Despite its lower prevalence, MIBC is typically associated with a relatively poor prognosis (5-year survival ranging from 30 to 70 %). The current gold-standard treatment, in the best of the cases, is the surgical removal of the bladder, with the subsequent impairment in quality of life, followed by radio-chemotherapy [1, 2].

RNA molecules of more than 200 nucleotides in length and a lack of protein-coding potential are collectively referred to as long non-coding RNAs (lncRNAs) [3]. Recently, increasing evidence suggests that lncRNAs regulate key pathways in cancer [4], constituting attractive novel prognostic markers and therapeutic targets [3]. Most lncRNAs function with DNA-binding proteins, such as chromatin-modifying complexes, and epigenetically regulate the expression of multiple genes [5].

LncRNA HOX antisense intergenic RNA(*HOTAIR*) is transcribed in the antisense direction from the HOXC gene cluster [6] and recruits Polycomb Repression Complex 2 (PRC2) to specific target genes genome-wide, leading to H3K27 trimethylation and epigenetic silencing of gene expression [7]. *HOTAIR* has been shown to be upregulated in cancer tissues and metastases, and its expression level correlates in general with metastasis and poor outcome in multiple cancer types [3–5, 7–10].

*ANRIL* is another example of lncRNA that is claimed as a participant directly in epigenetic transcriptional repression [11, 12]. *ANRIL* is transcribed from the INK4B-ARF-INK4A gene locus and binds to CBX7 [12], which belongs to Polycomb Repression Complex 1 (PRC1), to control the expression of p16INK4a, p15INK4b, and p14ARF tumor suppressor genes [13]. *ANRIL* also binds *SUZ12*, recruiting PRC2, and repressing the expression of the p15INK4B locus [14]. Based on these data, *ANRIL* would play dual roles, serving as a scaffold for both PRC1 and PRC2, as shown for *HOTAIR* [15].

Recently, we provided evidence from human patient samples and a novel transgenic mouse model that EZH2, the catalytic subunit of PRC2, mediates recurrence in NMIBC through gene expression modulation [16]. Here, we report the expression analysis of *HOTAIR* and *ANRIL*, which cooperate with PRC2 to allow specific gene repression and their possible roles as prognostic biomarkers in bladder cancer.

### Subjects and methods

#### Subjects

A series of 85 NMIBC patients has been analyzed and consecutively evaluated at the Urology Department of the University Hospital “12 de Octubre,” between October 2009 and December 2012 (Additional file 1: Table S1). Samples were collected by multiple cold-cup biopsies from the exophytic part of the tumor and from distant normal mucosa of the bladder of patients undergoing TUR. All samples were kept in RNAlater. Informed consent was obtained from all patients, and the study was approved by the Ethical Committee for Clinical Research of University Hospital “12 de Octubre.” Samples and united data from patients included in this study were provided by the Biobanco iþ12 in the Hospital 12 de Octubre integrated in the Spanish Hospital Biobanks Network (RetBioH; www.redbiobancos.es) following standard operation procedures with appropriate approval of the by the Ethical Committee for Clinical Research of University Hospital “12 de Octubre” (CEIC 10/50). The construction of a tissue microarray and analysis of EZH2 expression by immunohistochemistry has been reported elsewhere [16, 17].

#### RT-qPCR

Total RNA was isolated using miRNeasy Mini Kit (Qiagen) according to the manufacturer’s instructions, and DNA was eliminated (RNAse-Free DNAse Set Qiagen). Reverse transcription was performed from 10 ng of total RNA and using the Omniscript RT Kit (Qiagen) and specific primers for all genes of interest. PCR was performed in a 7500 Fast Real Time PCR System using Go Taq PCR master mix (Promega) and 1 μL of cDNA as a template. Melting curves were performed to verify specificity and absence of primer dimers. Reaction efficiency was calculated for each primer combination, and the *TBP* gene was used as the reference gene for normalization [18]. The sequences of the specific oligonucleotides used are listed in Additional file 1: Table S2.

#### Whole transcriptome analysis

Genome-wide transcriptome experiments using the Affymetrix HuGene-1_0-st-v1 microarray have been previously reported [16], and datasets have been deposited in GEO (GSE38264).

#### Cell cultures

Ten bladder cancer cell lines (Additional file 1: Table S3), kindly provided by Dr. FX Real (CNIO, Spain) [19], were maintained in DMEM GlutaMAX™ (Gibco-BRL Life Technologies) with 10 % fetal bovine serum (Hyclone) and 1 % antibiotic-antimycotic (Gibco-BRL Life Technologies) at 37 °C in a humidified atmosphere of 5 % CO2. Six different drugs (NVP-BEZ35 50 nM (LC Laboratories), Rapamycin 50 nM (LC Laboratories), Thyrphostin 100 μM (Sigma-Aldrich), Sb31542 10 nM (Sigma-Aldrich), DZNep 10 μM (Sigma-Aldrich), PD98059 10 μM (Sigma-Aldrich)) targeting key pathways (mTOR, Stat3, TGFβ, EZH2, and MAPK/ERK, respectively) were applied on a MGH-U4 non-muscle-invasive cell line for 6 h. In addition, DZNeP (10 μM) treatment was also applied at different time durations (0, 6, 24, and 48 h). For the knockdown of EZH2, the 5637 cell line was transduced with lentivirus-based shRNA (MISSION® shRNA, Sigma Aldrich) targeting human EZH2 gene (two independent shRNA constructs: TRCN0000353069 and TRCN0000286227). Cells were selected by puromycin (0.5 μg/mL; Sigma-Aldrich) resistance for 2 weeks, and pooled clones were collected. For the increased expression of EZH2, the RT112 cell line was transfected, using FuGENE®6 Transfection Reagent (Promega), with an EZH2-coding plasmid under CMV promoter. Transfected cells were selected by growing in the presence of hygromycin (250 μg/mL; Sigma-Aldrich), and pooled clones were collected.

#### Western blot

Pelleted cells were disrupted by freeze-thawing cycles in lysis buffer [200 mmol/L 4-(2-hydroxyethyl)-1-piperazineethanesulfonic acid, pH 7.9, 25 % glycerol, 400 mmol/L NaCl, 1 mmol/L EDTA, 1 mmol/L ethylene glycol tetraacetic acid, 1 mg/mL aprotinin, 1 mg/mL leupeptin, 1 mmol/L phenylmethylsulfonylfluoride, 20 mmol/L NaF, 1 mmol/L NaPPi, 1 mmol/L Na_3_VO_4_, 2.5 mmol/L dithiothreitol] and centrifuged to obtain supernatant containing total protein. Thirty-five micrograms of protein per sample was resolved in SDS-PAGE and transferred to nitrocellulose membranes (Amersham). Membranes were blocked with 0.1 % Tween-20 with 5 % bovine serum albumin (BSA) diluted in TBS and incubated with the appropriate antibodies diluted in TBS-T 0.5 % BSA. Secondary antibodies were purchased from Jackson Immuno Research. Super Signal West Pico Chemiluminescence Substrate (Pierce) was used according to the manufacturer’s recommendations to visualize the bands. Antibodies used are against EZH2 (Abnova MAB9542), AKT-P-Ser473 (Epitomics 2118-1), AKT-P-Thr308 (Cell Signaling 4056), and ERK-P-1/2 (Cell signaling 4370). Loading was controlled by using anti-GAPDH antibody (Santa Cruz sc-25778) and anti-ACTIN antibody (Santa Cruz sc-1616).

#### Statistical analysis

Comparisons were performed using the Wilcoxon-Mann-Whitney test (for two groups), Limma test, and the Student *t*-test for paired samples showing normal distribution. For multiple groups, the Kruskal-Wallis test followed by Dunn’s multiple comparison test was used. Correlations were calculated using Pearson correlation coefficient. Survival analyses (recurrence-free or overall survival), according to various variables, were performed using the Kaplan-Meier method, and differences between the patient groups were tested by the log-rank test. *p* values less than 0.05 were considered statistically significant. Discrimination between samples showing increased or decreased gene expression was made using the median. Overlapping significance was monitored by exact Fisher test. SPSS 17.0, R statistical software v2.15.1, and Graph prism5.0 software were used for processing of data. In the figures, *p* values are provided as follows: *p < 0.05; **p < 0.01; ***p < 0.005; ****p < 0.0001.

### Results and discussion

#### Expression of *HOTAIR* in NMIBC

**Fig. 1:**
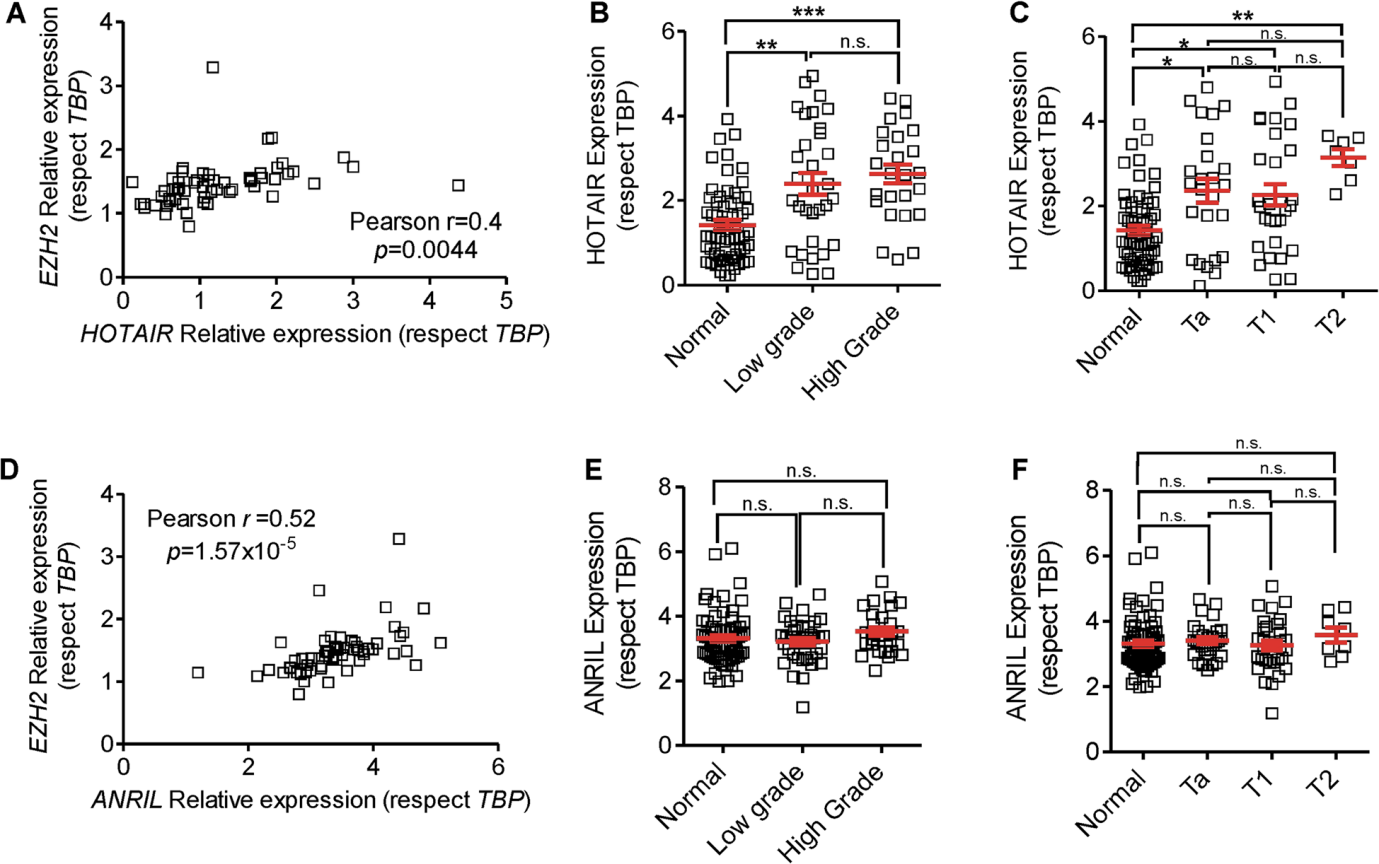
Expression of lncRNAs *HOTAIR* and *ANRIL* in NMIBC. **a** qPCR analyses showing the correlation between *EZH2* and *HOTAIR* expression in 64 NMIBC samples. **b**,**c** qPCR analyses showing the expression of *HOTAIR* in normal and NMIBC samples according the tumor grade (*b*) and stage (*c*). **d** qPCR analyses showing the correlation between *EZH2* and *ANRIL* expression in 64 NMIBC samples. **e,f** qPCR analyses showing the expression of *ANRIL* in normal and NMIBC samples according the tumor grade (*e*) and stage (*f *). Correlations were calculated using Pearson correlation coefficient. Comparisons between gene expression levels were done using the Kruskal-Wallis test followed by Dunn’s multiple comparison. *p* values are provided as follows: *p < 0.05; **p < 0.01; ***p < 0.005. *TBP* was used as normalizer gene [18]

High expression levels of *HOTAIR* have been proven to correlate with metastasis and poor prognosis in many types of cancer [15]. However, its role during bladder oncogenesis is still to be clarified. In the present study, we determined the gene expression levels of *HOTAIR*, PRC2 members (*EZH2*, *SUZ12*, *EED*), and *BMI1* (PRC1) by qPCR in a series of NMIBC patients (*n* = 64). We observed a significant and positive correlation between *HOTAIR* and all PRC2 members and *BMI1* (Fig. 1a and Additional file 2: Figure S1A–C). Regarding the *HOTAIR* gene levels, we observed that compared to normal paired samples its levels were increased in tumors regardless their different grades (Fig. 1b) or stages (Fig. 1c). This finding is in agreement with other cancers [4, 5, 20, 21] and with a recently reported study of bladder cancer from a Chinese population [22].

#### Expression of *ANRIL* in NMIBC

No data are available on the possible role of *ANRIL* in BC. Since it mediates silencing p15INK4b by binding to *SUZ12* [14], and p16INK4a locus through *CBX7* (a PRC1 component) recruitment [12], which is frequently silenced in BC [23], we analyzed its expression in our patient dataset. The qPCR data revealed, as in the case of *HOTAIR*, a significant positive correlation between *ANRIL* and all PRC2 members and *BMI-1* (Fig. 1d and Additional file 2: Figure S1D–F). However, no differences were found between normal and tumor samples regardless their grade (Fig. 1e) or stage (Fig. 1f).

#### *HOTAIR* mediates recurrence and progression in NMIBC

The high frequency of recurrence is a current clinical problem in NMIBC. To analyze whether *ANRIL* or *HOTAIR* expression could be a recurrence determinant, we characterized their expression in primary tumors that have subsequently developed recurrence or not after a follow-up period (Additional file 1: Table S1). In the case of *ANRIL*, its expression levels could not discriminate between recurrent and non-recurrent tumors (Additional file 3: Figure S2). This lack of correlation, together with the previous results, showing no major differences in normal and tumor samples or according tumor clinicopathological characteristics, supports that this lncRNA does not seem to have a major role in NMIBC.

**Fig. 2:**
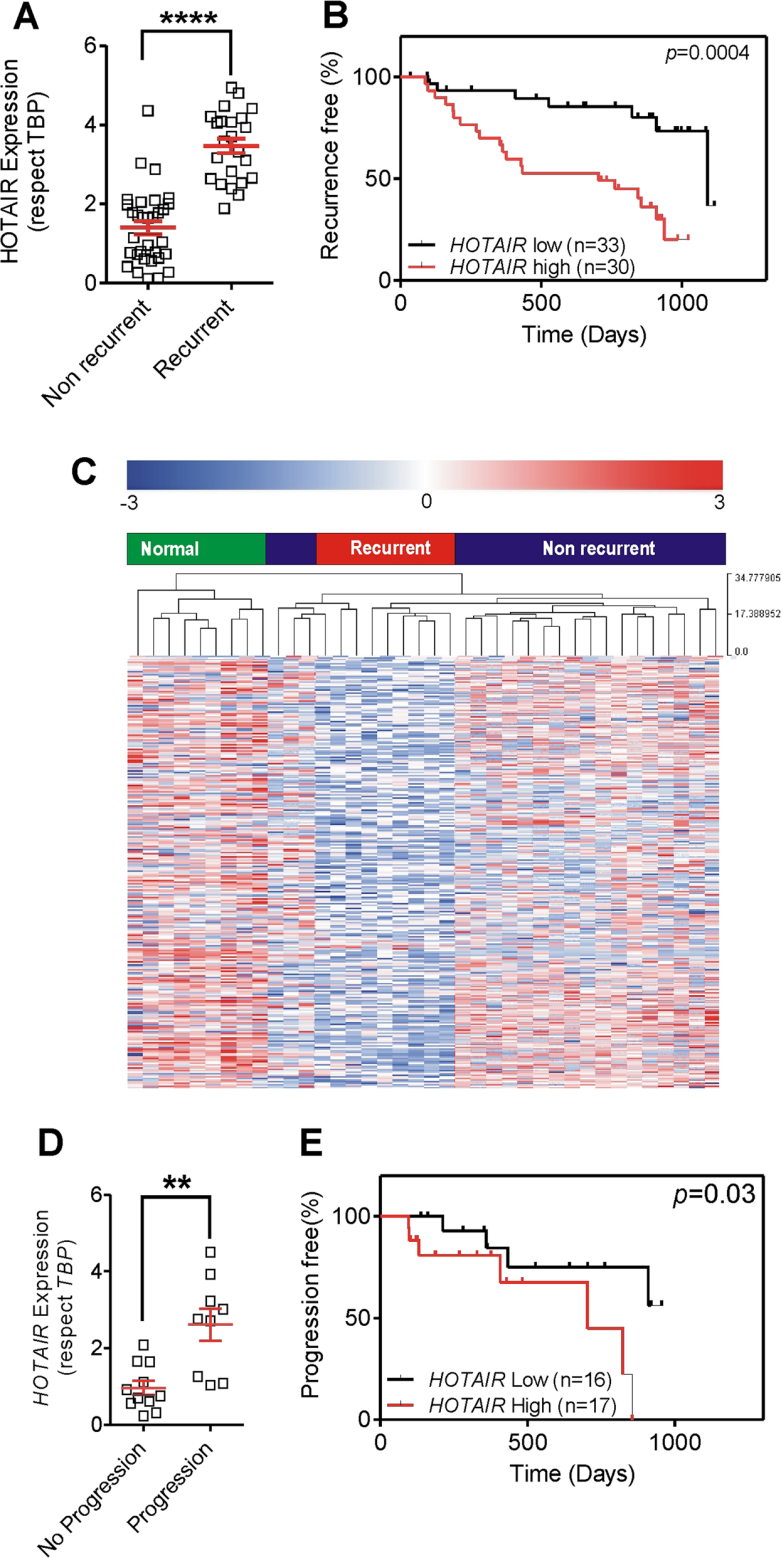
*HOTAIR* mediates recurrence and progression in NMIBC. **a** qPCR analyses showing the expression of *HOTAIR* in NMIBC samples according the tumor recurrence. **b** Kaplan-Meier analysis showing that patients with higher *HOTAIR* expression (according the median) showed an earlier recurrence (*p* value was obtained by the log-rank test). **c** Heat map showing the distribution of genes (*rows*) and samples following unsupervised clustering (Pearson correlation and average linkage method) of 28 tumors and 10 normal samples [16] according the expression of genes previously identified by the binding of *HOTAIR-EZH2* complexes [7, 24]. A *red* (overexpressed) to *blue* (downregulated) scheme following the above scale limits (in log_2_ scale) is shown. Note that recurrent and non-recurrent tumors, besides of non-tumor bladder tissue, could be efficiently discriminated. **d** qPCR analyses showing the expression of *HOTAIR* in recurrent NMIBC samples according the tumor progression. **e** Kaplan-Meier analysis showing that patients with higher *HOTAIR* expression (according the median) showed an earlier progression upon recurrence (*p* value was obtained by the log-rank test)

Interestingly, recurrent tumors showed a significantly higher *HOTAIR* expression in comparison with non-recurrent tumors (Fig. 2a). In fact, when patients were stratified according high or low *HOTAIR* levels (above or below median), those patients with higher *HOTAIR* expression exhibited significantly earlier recurrence (Fig. 2b).

*HOTAIR* recruits the PRC2 complex to specific target genes, which leads to H3K27 trimethylation and epigenetic silencing, and a large number of genes have been previously identified by the binding of *HOTAIR*-*EZH2* complexes [7, 24]. We thus used this gene dataset in our previous genome-wide transcriptome data of human NMIBC samples [16]. Remarkably, the expression of the *HOTAIR*-*EZH2* target genes could efficiently discriminate between recurrent and non-recurrent tumors, as well as non-tumor bladder tissue in an unsupervised manner (Fig. 2c). These results reinforce that the altered gene expression programs displayed by NMIBC with a high-recurrence probability are mediated by *HOTAIR-EZH2* complexes. Therefore, our current results indicated that *HOTAIR* might be a potential prognostic biomarker for recurrence.

Tumor progression, defined as an increased tumor stage or grade in recurrences, is a common clinical problem in the management of BC. In our series, 11 out of 33 patients (Additional file 1: Table S1) displayed tumor progression. To analyze whether increased *HOTAIR* expression could define a progression biomarker, we determined the *HOTAIR* levels in recurrent samples according to the tumor progression. We found that primary tumors in patients displaying tumor progression were characterized by increased *HOTAIR* levels (Fig. 2d). Similarly, those patients with high *HOTAIR* expression exhibited significantly (*p* = 0.03) earlier progression in recurrences (Fig. 2e).

#### *HOTAIR* expression in MIBC

**Fig. 3:**
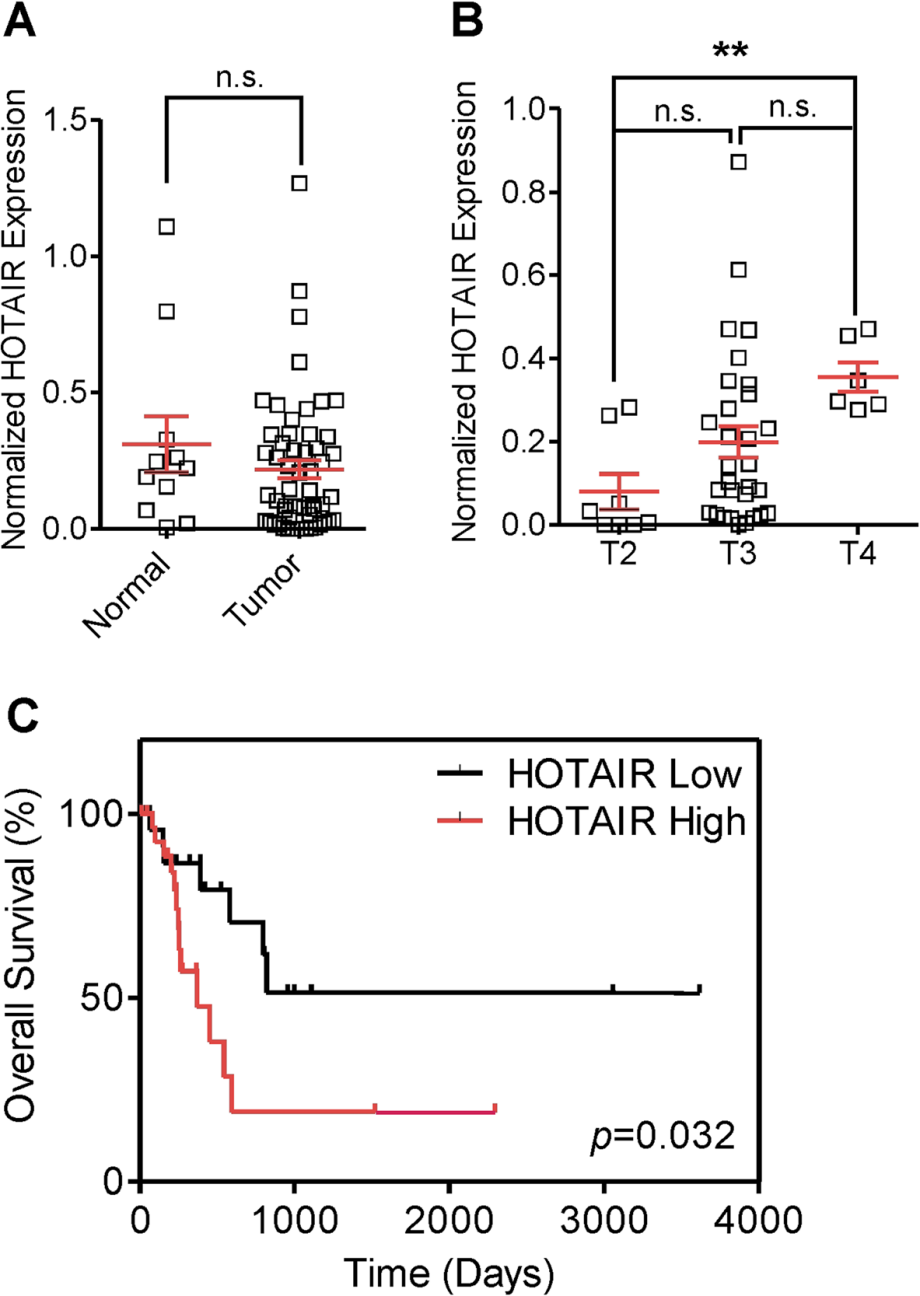
*HOTAIR* expression in MIBC from the TCGA portal. **a** Expression of *HOTAIR* in normal and MIBC tumor samples. Comparison was performed by Limma test. **b** Expression of *HOTAIR* in MIBC samples according tumor stage. Comparison was performed by Limma test. **c** Kaplan-Meier analysis of MIBC patient overall survival according *HOTAIR* expression (median discrimination) (*p* value was obtained by the log-rank test). RNA-seq data were downloaded from the TCGA portal (https://tcga-data.nci.nih.gov/tcga/)

Since in our patient sample series (see Additional file 1: Table S1) there is a higher representation of non-muscle-invasive bladder cancer samples (87 %) compared to T2 samples (*n* = 11), we also prompted to analyze any potential roles of *HOTAIR* expression in muscle-invasive (T2–4) tumors. To this, we have used the RNA-seq data available at the TCGA Data Portal (https://tcga-data.nci.nih.gov/tcga/). We observed no significant differences between normal and tumor samples (Fig. 3a), probably due to the low representation of normal bladder samples in this dataset. The comparison between grades was not possible since all the samples available for RNA-seq analyses  with clinical information are high grade. In the case of stages, a significant increase (*p* = 0.006) in *HOTAIR* expression was found according to cancer progression (Fig. 3b), finding higher levels when the cancer spreads across muscle layers (T2 stage) to the surrounding organs (T4). These results suggest that *HOTAIR* is a potential epigenetic marker of the molecular stages of bladder cancer. Finally, the Kaplan-Meier analysis showed that patients who had higher *HOTAIR* expression (above median) had significantly shorter overall survival than those with lower expression (Fig. 3c). This finding indicates that *HOTAIR* could represent a powerful independent prognostic factor of malignant progression, recurrence, and overall survival rate in BC. Similar findings have been recently reported for several other types of cancer [4, 5, 7, 8, 20, 21].

#### The role of methylation

Since methylation of *HOTAIR* has already been reported [25], we wondered if these differences in *HOTAIR* expression could be due to differences in *HOTAIR* methylation. Using Illumina HumanMethylation450 Array data from the TCGA portal, no relevant DNA methylation differences were found between normal and tumor samples along the *HOTAIR* gene body (Additional file 4: Figure S3A). Using the same system, when normal and tumor samples were checked in a different patient dataset, including our low-grade samples and high-grade samples from UCL (personal communication), again no significant differences were found (Additional file 4: Figure S3B). Similarly, the comparison between low- and high-grade tumor samples showed no significant differences in *HOTAIR* methylation; the differences are even smaller (Additional file 4: Figure S3C). These results indicate that DNA methylation, at least regarding the assayed sites, cannot determine the differences in *HOTAIR* expression between normal tissue and tumors or between low- and high-grade tumors.

#### Functional evidence of association between EZH2 and *HOTAIR* expression

The precise regulatory mechanisms affecting *HOTAIR* expression are still largely unknown. The positive correlation observed between *EZH2* and *HOTAIR* levels (Fig. 1a) might indicate a co-regulatory process. Similarly, we observed a trend of positive correlation in samples showing high EZH2 protein expression (Additional file 5: Figure S4).

**Fig. 4:**
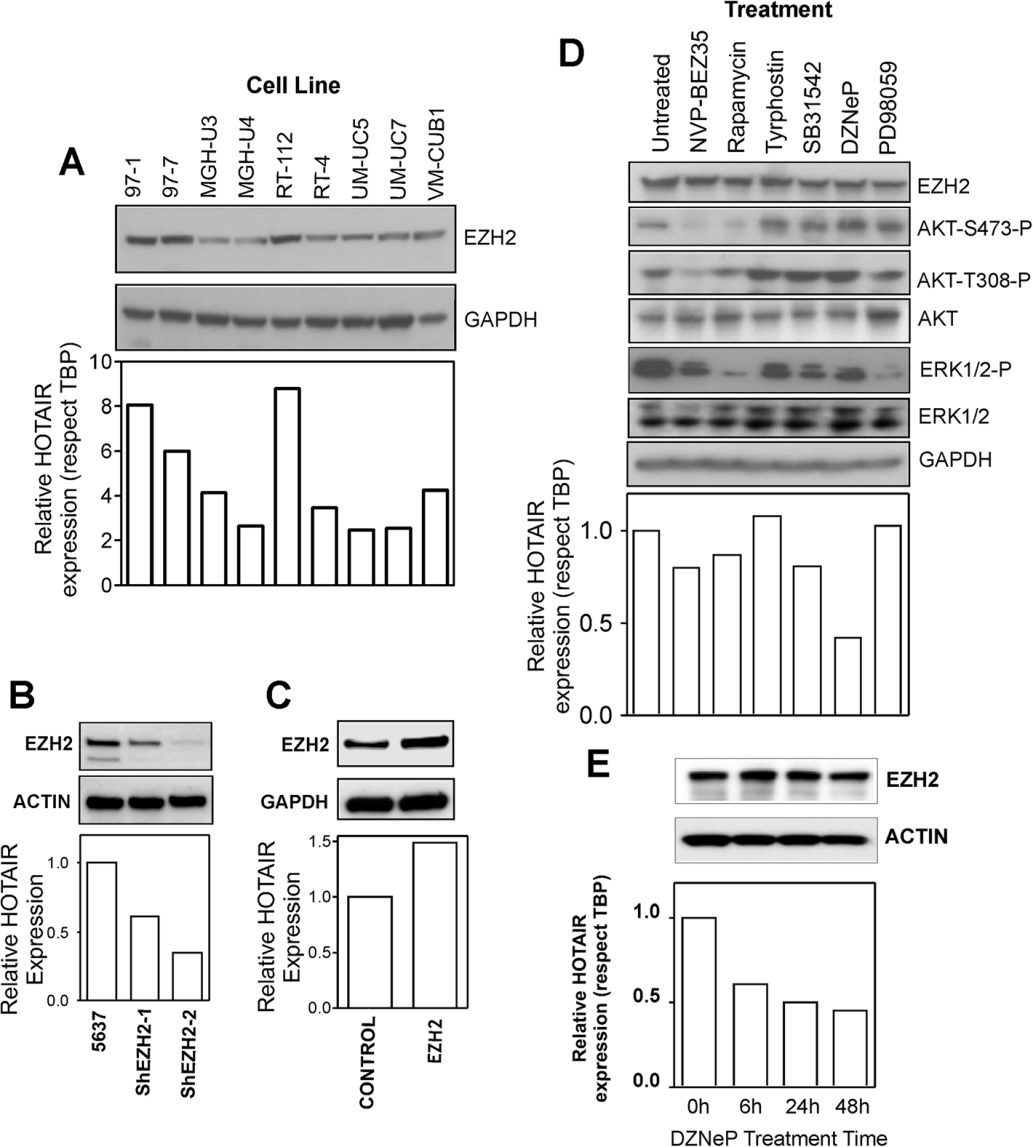
Functional evidence of *HOTAIR* regulation by EZH2. **a** Expression of EZH2 protein as assessed by immunoblot in the quoted non-invasive bladder cancer cell lines in parallel with *HOTAIR* (bar graph, assessed by RT-qPCR). **b** Expression of EZH2 protein (immunoblot) and *HOTAIR* (bar graph) in 5637 MIBC cell lines upon knockdown mediated by two different shRNA constructs. **c** Expression of EZH2 protein (immunoblot) and *HOTAIR* (bar graph) in RT112 NMIBC cell line upon transfection with CMV-EZH2-coding plasmid. **d** Expression of the quoted proteins and *HOTAIR* (bar graph) in MGH-U4 NMIBC cell line upon treatment (24 h) with NVP-BEZ35 (50 nM), rapamycin (50 nM), tyrphostin (100 μM), SB31542 (10 nM), DZNeP (10 μM), and PD98059 (10 μM). Note that *HOTAIR* expression is only significantly reduced upon treatment with the EZH2-specific inhibitor DZNeP. **e** Expression of EZH2 protein (immunoblot) and *HOTAIR* (bar graph) in MGH-U4 NMIBC cell line upon treatment for different time periods with DZNeP (10 μM). GAPDH and ACTIN were used for loading control in immunoblots, and *TBP* was used as a normalizer gene for RT-qPCR

The analysis of *HOTAIR* and EZH2 expression in nine different non-invasive bladder cancer cell lines also showed that those cells showing high EZH2 protein levels also displayed high *HOTAIR* expression (Fig. 4a), thus reinforcing their co-regulation. To test whether EZH2 could modulate *HOTAIR* expression, we performed knockdown of EZH2 in 5637 cells (a MIBC cell line showing the highest EZH2 expression; not shown). We observed that the reduction of EZH2 is accompanied with a substantial reduction of *HOTAIR* levels (Fig. 4b). Similarly, the increased expression of EZH2 by transfection of RT112 cells is also in parallel with increased expression of HOTAIR (Fig. 4c). These findings indicate that EZH2 could regulate the expression of *HOTAIR*.

We next studied the effect of various pharmacological inhibitors (described in the “Subjects and methods” section) of several key cancer signal transduction pathways in the MGH-U4 cell line. We observed that only the EZH2-specific inhibitor DZNep [26, 27] produced a significant decrease in *HOTAIR* expression (Fig. 4d). In agreement, the treatment with DZNep for different time periods within these cells, revealed that 6 h of treatment, was sufficient to lower *HOTAIR* levels (Fig. 4e). These results suggest that the activity of EZH2 is required to modulate *HOTAIR* expression. However, whether this regulation is executed directly or indirectly remains to be elucidated in the future.

### Conclusions

Differential regulation of long non-coding RNAs has been reported in different types of cancer although their functional mechanisms are still unknown and intriguing. Our experiments show a poor involvement of *ANRIL* in BC, while confirming an upregulation of *HOTAIR* gene expression levels in recurrent and high-graded tumors associated with a poor prognosis, in both NMIBC and MIBC. In this context, we have recently shown that EZH2 mediates recurrence in NMIBC, being also upregulated in tumor samples and especially in recurrent tumors [16].The current study gives an insight into the regulation of *HOTAIR*, indicating that EZH2 may regulate *HOTAIR* expression. This finding might have therapeutic implications for the treatment of recurrent NMIBC, since there are several EZH2 inhibitors in preclinical studies that can be of interest as therapeutic tools in tumors showing increased *HOTAIR* expression. The present data also indicate that such inhibition may result in decreased *HOTAIR* levels, favoring the rewiring of gene expression as a possible therapeutic strategy.

## Additional files

Additional file 1: Table S1. Table S2. Table S3. Baseline Characteristics of the patients and Clinical and pathological results in the series. **Table S2.** Oligo sequences. **Table S3.** Cell Lines Used. (DOCX 31 kb) Additional file 2: Figure S1. Correlation between *HOTAIR* and *ANRIL* with all PRC2 members and *BMI-1*. (PDF 124 kb) Additional file 3: Figure S2. Expression of *ANRIL* in non recurrent and recurrent tumor samples. (JPEG 139 kb) Additional file 4: Figure S3. Methylation along the *HOTAIR* gene body. (PDF 329 KB) Additional file 5: Figure S4. Expression of *HOTAIR *in samples with positive and negative staining in TMA. (JPEG 152 KB)
