# Supplementary material for: Analysis of the Polycomb-related lncRNAs HOTAIR and ANRIL in bladder cancer
Source: Clin Epigenetics. 2015 Oct 8;7:109. doi: 10.1186/s13148-015-0141-x (PMC4599691; doi:10.1186/s13148-015-0141-x)
Supplement: Additional file 1: Table S1. Table S2. Table S3. — Baseline Characteristics of the patients and Clinical and pathological results in the series. Table S2. Oligo sequences. Table S3. Cell Lines Used. (DOCX 31 kb) [file 13148_2015_141_MOESM1_ESM.docx]

**Additional file 1**

**Supplementary Table I**

**Baseline Characteristics of the patients and Clinical and pathological results in the series**

| Patients (n) | 85 |
| --- | --- |
| Age median (range) | 73 yr (49-90) |
| Sex | M=66  F=19 |
| Stage | Ta=38  T1=36  T2=11 |
| Grade | Low=44  High=35  ^a^PUNLMP =5 |
| Alterations in normal mucosa | Dysplasia=6 |
| Follow up (days), median (range) | 887d (34-1135) |
| Tumor size, median (range) | 2.5cm (0.5-7) |
| Number of implants, median (range) | 1 (1-10) |
| Specific death events^b^ | 8 |
| Non-specific deaths^c^ | 3** |
| Recurrence events^d^ | No recurrence =37  Recurrence =42 |
| Time to recurrence, median (range) | 659d (34-1113) |
| Progression in recurrence^e^ | 11 |

a) PUNLMP Papillary Urothelial neoplasia of low malignant potential

b) All deaths due to progression of invasive bladder cancer.

c) One patient died due to a lymphoma, another of sepsis secondary to mycosis fungoides, another due to progression of gastric tumor and another of chronic obstructive pulmonary disease decompensation

d) Only NMIBC patients are considered

e) Recurrent tumors display increased stage and/or grade

**Supplementary Table II**

**Oligo sequences**

ANRIL RTsp: 5’-CCAAGTCTGGACTGTGAGACA-3’

F 5’-CAACATCCACCACTGGATCTTAACA-3’

R 5’-AGCTTCGTATCCCCAATGAGATACA-3’

HOTAIR RTsp: 5’-GGGTGTTGGTCTGTGGAACT-3’

F 5’- CAGTGG-GGAACTCTGACTCG-3’

R 5’-GTGCCTGGTG-CTCTCTTACC-3’

EZH2 RTsp: 5’-CCTGTCGACATGTTTTGGTC-3’

F 5’- CGGTGTCAAACGCCAATAAA-3’

R 5’- CCGCTCCACTCCACATTCTC-3’

EED RTsp: 5’-TGCTTCCTACAGTTGCAAACAC-3’

F 5’- GAACGCCCTGATACACCTACAAA-3’

R 5´- CATTTTCCCTTCCCCCAACT-3’

SUZ 12 RTsp: 5’-CTCTTCTTCCTGGACGAGTCA-3’

F 5’- TCCGATAAGGCAAGTTCCTACAG-3’

R 5’- GAAGTTTCCAGGTTTTGTTTGATTG-3’

BMI-1 RTsp: 5’- ATGGTTGTGGCATCAATGAA-3’

F 5’- GCTTCAAGATGGCCGCTT G-3’

R 5’- TTCTCGTTGTTCGATGCATTT C-3’

TBP RTsp: 5’-GTGTTTAAAATCTACATA-3’

F: 5’-AGTGAAGAACAGTCCAGACTG-3’

R: 5’-CCAGGAAATAACTCTGGCTCAT-3’

**Suplementary Table III**

**Cell Lines Used**

| **Name** | **Origin**  **(Stage and Grade)** | **Reference** |
| --- | --- | --- |
| 97-1 | T1/T2; G1/G2 | ([Yeager, DeVries et al. 1998](#_ENREF_23)) |
| 97-7 | T1; G3 | ([Sarkar, Julicher et al. 2000](#_ENREF_21)) |
| MGH-U3 | Ta; G1 | ([Lin, Lin et al. 1985](#_ENREF_12)) |
| MGH-U4 | Papilloma; G1 | ([Lin, Lin et al. 1985](#_ENREF_12)) |
| RT112 | Unknown; G2 | ([Masters, Hepburn et al. 1986](#_ENREF_13)) |
| RT 4 | T2; G1 | ([Rigby and Franks 1970](#_ENREF_19); [O'Toole, Povey et al. 1983](#_ENREF_16); [Masters, Hepburn et al. 1986](#_ENREF_13)) |
| UMU-UC5 | Unknown; Unknown | ([Grossman, Wedemeyer et al. 1986](#_ENREF_8)) |
| UMU-UC7 | Unknown; Unknown | ([Grossman, Wedemeyer et al. 1986](#_ENREF_8)) |
| VM-CUB1 | Unknown; G2 | ([Fogh 1978](#_ENREF_5); [Masters, Hepburn et al. 1986](#_ENREF_13)) |
| 5637 | T2;G2 | ([Fogh, Fogh et al. 1977](#_ENREF_6); [Rieger, Little et al. 1995](#_ENREF_18)) |
