# Supplementary material for: Analysis of the Polycomb-related lncRNAs HOTAIR and ANRIL in bladder cancer
Source: Clin Epigenetics. 2015 Oct 8;7:109. doi: 10.1186/s13148-015-0141-x (PMC4599691; doi:10.1186/s13148-015-0141-x)
Supplement: Additional file 2: Figure S1. — Correlation between HOTAIR and ANRIL with all PRC2 members and BMI-1. (PDF 124 kb) [file 13148_2015_141_MOESM2_ESM.pdf]

Additional file 2: Figure S1

Correlation between *HOTAIR* and *ANRIL* with all PRC2 members and *BMI-1*

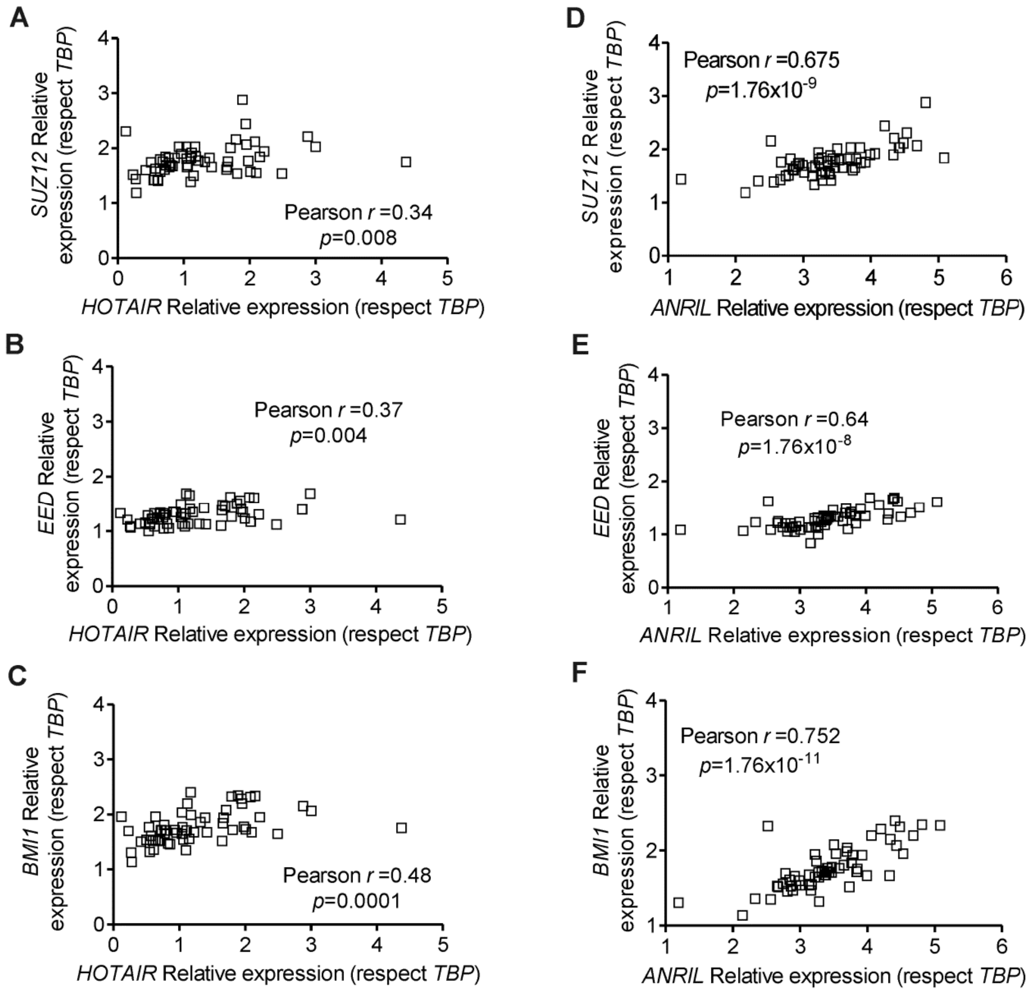

A) qPCR analyses showing the correlation between *SUZ12* and *HOTAIR* expression

B) qPCR analyses showing the correlation between *EED* and *HOTAIR* expression

C) qPCR analyses showing the correlation between *BMI-1* and *HOTAIR* expression

D) qPCR analyses showing the correlation between *SUZ12* and *ANRIL* expression

E) qPCR analyses showing the correlation between *EED* and *ANRIL* expression

F) qPCR analyses showing the correlation between *BMI-1* and *ANRIL* expression

Correlations were calculated using Pearson correlation coefficient. TBP was used as normalizer gene.
