# Supplementary figures and images for: Analysis of the Polycomb-related lncRNAs HOTAIR and ANRIL in bladder cancer
Source: Clin Epigenetics. 2015 Oct 8;7:109. doi: 10.1186/s13148-015-0141-x (PMC4599691; doi:10.1186/s13148-015-0141-x)

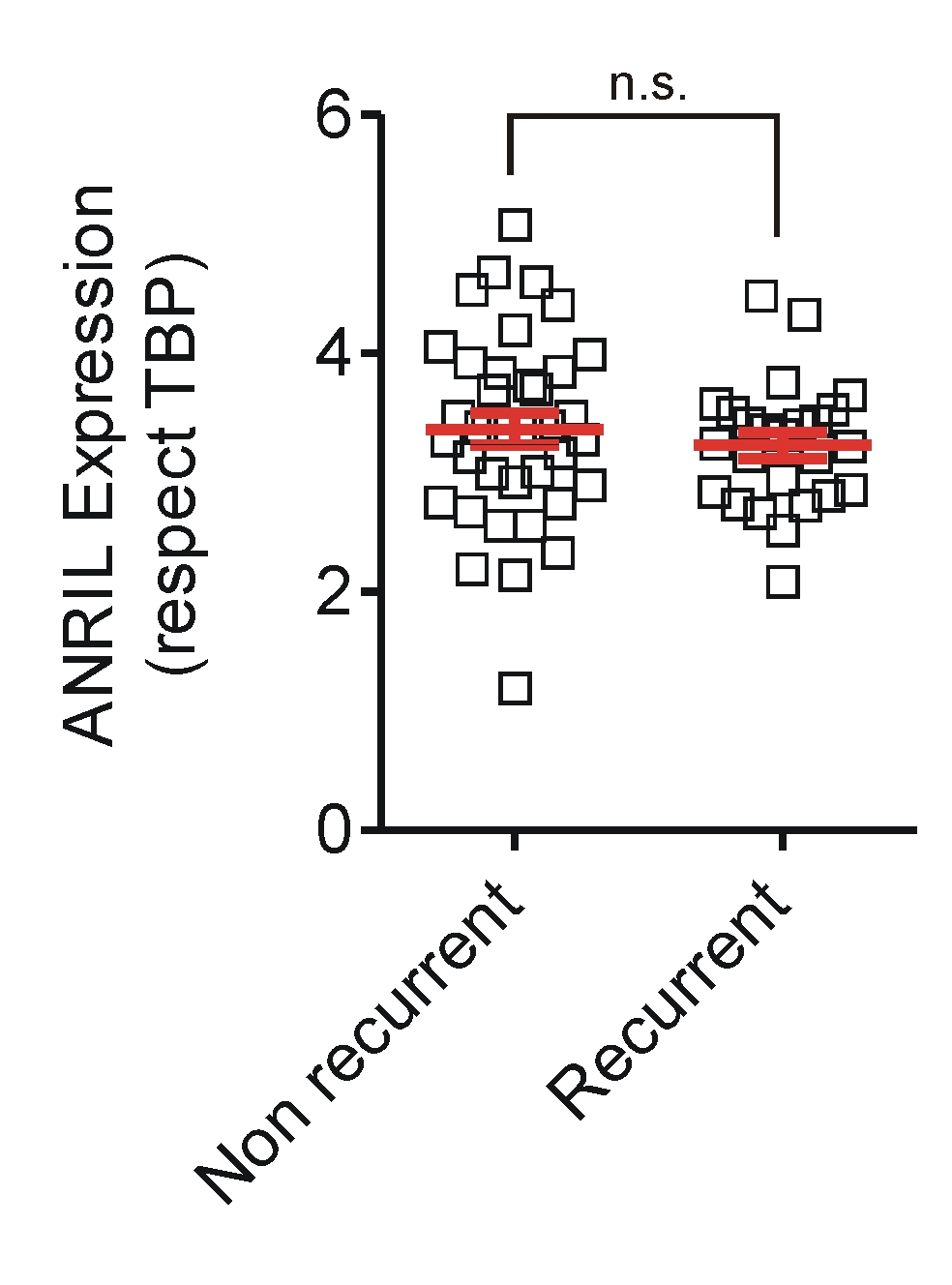

Supplement: Additional file 3: Figure S2. — Expression of ANRIL in non recurrent and recurrent tumor samples. (JPEG 139 kb) [file 13148_2015_141_MOESM3_ESM.jpeg]

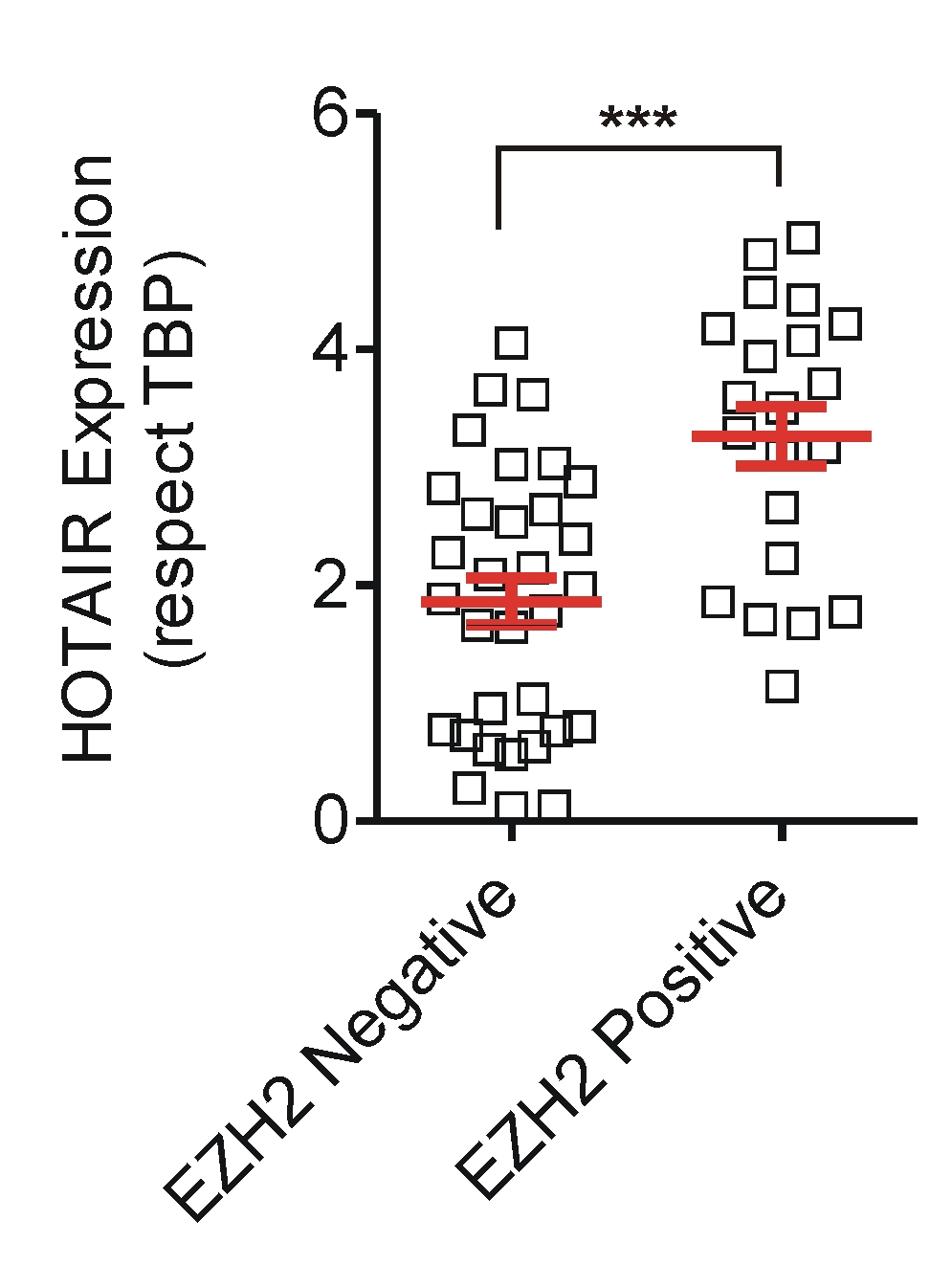

Supplement: Additional file 5: Figure S4. — Expression of HOTAIR in samples with positive and negative staining in TMA. (JPEG 152 KB) [file 13148_2015_141_MOESM5_ESM.jpeg]
