# Supplementary material for: Analysis of the Polycomb-related lncRNAs HOTAIR and ANRIL in bladder cancer
Source: Clin Epigenetics. 2015 Oct 8;7:109. doi: 10.1186/s13148-015-0141-x (PMC4599691; doi:10.1186/s13148-015-0141-x)
Supplement: Additional file 4: Figure S3. — Methylation along the HOTAIR gene body. (PDF 329 KB) [file 13148_2015_141_MOESM4_ESM.pdf]

Additional file 4: Figure S3

Methylation along the *HOTAIR* gene body

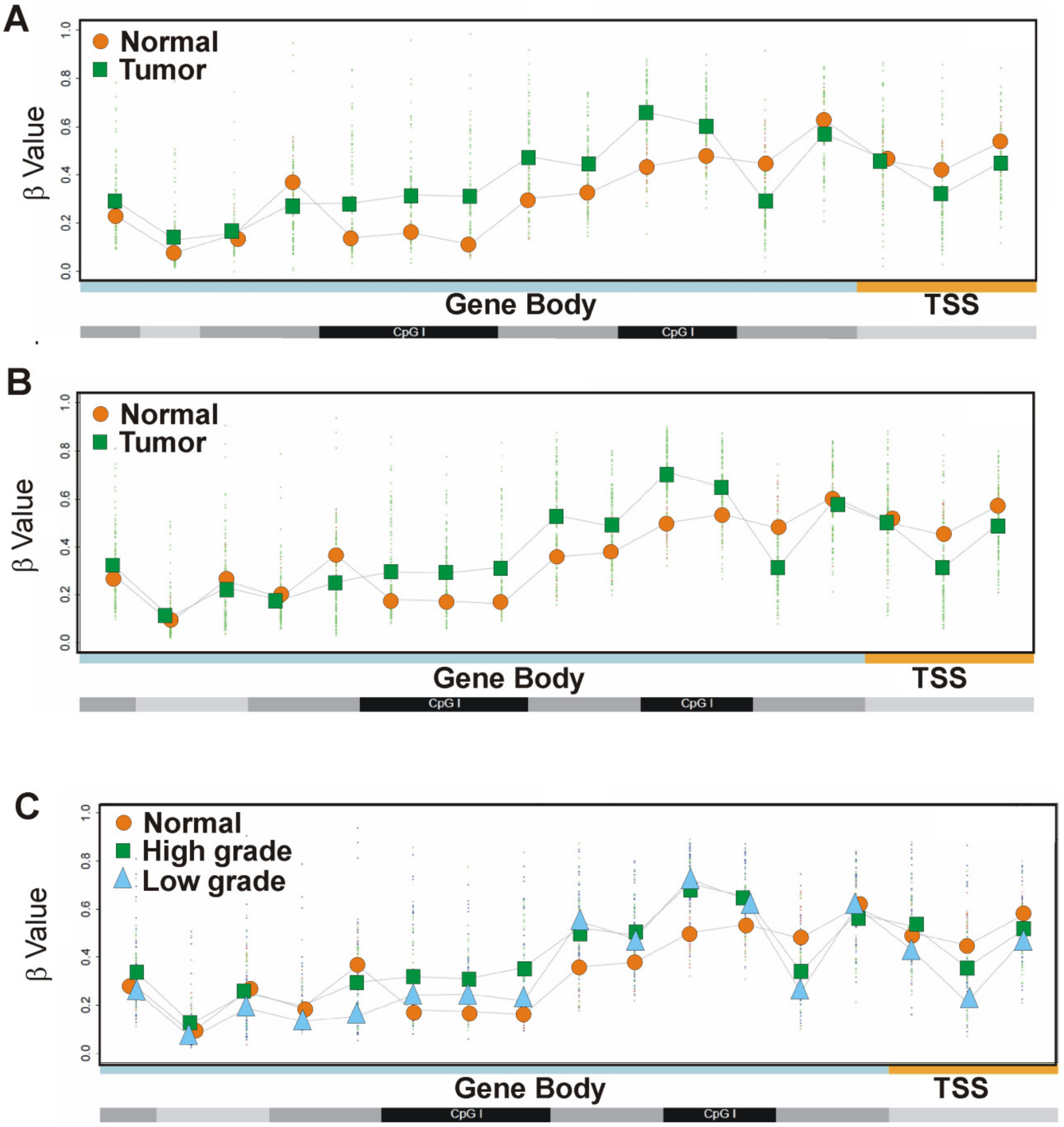


A) Differences in methylation between normal and MIBC from TCGA portal along the *HOTAIR* gene body using Illumina HumanMethylation450 Array

B) Differences in methylation between normal and BC samples (both MIBC and NMIBC) along the *HOTAIR* gene body using Illumina HumanMethylation450 Array

C) Differences in methylation between normal and both high grade (UCL) and low grade (CIEMAT) along the *HOTAIR* gene body using Illumina HumanMethylation450 Array

TSS: transcription Start Site

CpG I: position of the CpG Islands
